# Probiotic properties of *Bacillus subtilis* DG101 isolated from the traditional Japanese fermented food nattō

**DOI:** 10.3389/fmicb.2023.1253480

**Published:** 2023-09-28

**Authors:** Cecilia Leñini, Facundo Rodriguez Ayala, Anibal Juan Goñi, Liliana Rateni, Akira Nakamura, Roberto Ricardo Grau

**Affiliations:** ^1^Departamento de Microbiología, Consejo Nacional de Investigaciones Científicas y Técnicas (CONICET), Facultad de Ciencias Bioquímicas y Farmacéuticas, Universidad Nacional de Rosario, Rosario, Argentina; ^2^Faculty of Life and Environmental Sciences, University of Tsukuba, Tsukuba, Japan

**Keywords:** probiotics, fermented soybean, nattō, *Bacillus subtilis* DG101, beneficial biofilms, metal bioremediation, gut health

## Abstract

Spore-forming probiotic bacteria offer interesting properties as they have an intrinsic high stability, and when consumed, they are able to survive the adverse conditions encountered during the transit thorough the host gastrointestinal (GI) tract. A traditional healthy food, nattō, exists in Japan consisting of soy fermented by the spore-forming bacterium *Bacillus subtilis* natto. The consumption of nattō is linked to many beneficial health effects, including the prevention of high blood pressure, osteoporosis, and cardiovascular-associated disease. We hypothesize that the bacterium *B. subtilis* natto plays a key role in the beneficial effects of nattō for humans. Here, we present the isolation of *B. subtilis* DG101 from nattō and its characterization as a novel spore-forming probiotic strain for human consumption. *B. subtilis* DG101 was non-hemolytic and showed high tolerance to lysozyme, low pH, bile salts, and a strong adherence ability to extracellular matrix proteins (i.e., fibronectin and collagen), demonstrating its potential application for competitive exclusion of pathogens. *B. subtilis* DG101 forms robust liquid and solid biofilms and expresses several extracellular enzymes with activity against food diet-associated macromolecules (i.e., proteins, lipids, and polysaccharides) that would be important to improve food diet digestion by the host. *B. subtilis* DG101 was able to grow in the presence of toxic metals (i.e., chromium, cadmium, and arsenic) and decreased their bioavailability, a feature that points to this probiotic as an interesting agent for bioremediation in cases of food and water poisoning with metals. In addition, *B. subtilis* DG101 was sensitive to antibiotics commonly used to treat infections in medical settings, and at the same time, it showed a potent antimicrobial effect against pathogenic bacteria and fungi. In mammalians (i.e., rats), *B. subtilis* DG101 colonized the GI tract, and improved the lipid and protein serum homeostasis of animals fed on the base of a normal- or a deficient-diet regime (dietary restriction). In the animal model for longevity studies, *Caenorhabditis elegans*, *B. subtilis* DG101 significantly increased the animal lifespan and prevented its age-related behavioral decay. Overall, these results demonstrate that *B. subtilis* DG101 is the key component of nattō with interesting probiotic properties to improve and protect human health.

## Introduction

Probiotics are live microorganisms that produce beneficial effects on the host’s health when consumed in the recommended amounts (Raya et al., 2002; Hill et al., 2014). One essential attribute of a probiotic is the proficiency to arrive active (i.e., alive) at the site of action (e.g., the gastrointestinal, GI, tract) (Binda et al., 2020). Typical probiotics are represented by lactic acid bacteria (LAB), such as certain strains belonging to the genus *Lactobacilli, Streptococci* and *Bifidobacterium* (Haro et al., 2018; Lemme-Dumit et al., 2018). The beneficial properties of probiotic LAB on humans have some limitations related to the LAB lability and susceptibility to environmental stressors (e.g., temperature changes, dehydration, and the hostile gut environment) (Duary et al., 2011; Pedraza-Reyes et al., 2012; Botta et al., 2014; Gomathi et al., 2014). Other types of human probiotic bacteria that successfully overcome these concerns are represented by spore-forming bacteria of the *Bacillus* genus (e.g., *Bacillus subtilis*, *Bacillus coagulans*, and *Bacillus clausii*) (Sanders et al., 2003; Kalman et al., 2009; Cutting, 2011; Hanifi et al., 2015; Piewngam et al., 2018; Piewngam and Otto, 2020; Freedman et al., 2021; Rodriguez Ayala et al., 2021; Zeng et al., 2021). These probiotic bacilli possess the ability to form resistant spores protecting them from hostile environmental conditions and preserving their viability as probiotics (De Vecchi and Drago, 2006; Hong et al., 2008; Cutting, 2011; Binda et al., 2020). Interestingly, recent reports have shown the compatibility between probiotic LAB and bacilli, with a focus on the protective role of the bacilli biofilm on LAB survival and functionality (Zhang et al., 2013; VidyaLaxme et al., 2014; Yang et al., 2015; Yahav et al., 2018).

In Asian and African countries, there are several bacilli-fermented foods that produce beneficial effects on human health (Dimidi et al., 2019). One of these healthy fermented foods is nattō, a traditional Japanese food made from whole soybeans that have been fermented with a specific variety of *B. subtilis* (i.e., *B. subtilis* var. natto) (Sumi et al., 1987; Ueda, 1989; Fujita et al., 1993; Osawa and Matsumoto, 1997; Fujita et al., 2022). *B. subtilis* natto produces several molecules associated with human health (Su et al., 2020), including large amounts of vitamin K2 (menaquinone-7), important for bone robustness (Ikeda and Doi, 1990; Tsukamoto et al., 2000; Hu et al., 2020; Wu et al., 2021); pyrroloquinoline quinone (PQQ), a natural antioxidant with energizing and anti-fatigue effects (Nakano et al., 2012, 2015); nattokinase, a protease conferring protection against high blood pressure (Sumi et al., 1987; Fujita et al., 1993; Kim et al., 2008), ischemic stroke (Urano et al., 2001; Hsu et al., 2009; Murakami et al., 2012; Ji et al., 2014; Pham et al., 2020; Suzuki et al., 2023), cardiovascular disease (Urano et al., 2001; Hsu et al., 2009; Murakami et al., 2012; Weng et al., 2017; Suzuki et al., 2023), with anti-neurodegenerative (Hsu et al., 2009; Kumar et al., 2017) and anti-celiac effects (Wei et al., 2016); and cutaneous wound healing properties (Zhang et al., 2019). In addition, *B. subtilis* produces natural antimicrobials (lipopeptide antibiotics) that demonstrated activity against pathogenic bacteria (Ongena et al., 2005; Cao et al., 2009; Piewngam et al., 2018; Piewngam and Otto, 2020), fungi (Yu et al., 2002; Cawoy et al., 2015; Bartolini et al., 2019), viruses (Itokawa et al., 1994; Vollenbroich et al., 1997; Jung et al., 2000), and cancer cells (Nagata et al., 2002; Wang et al., 2007; Cao et al., 2009; Myung et al., 2009; Zhang et al., 2015; Lu et al., 2017). Therefore, it is not surprising that the regular consumption of nattō (and other soy-fermented foods) is linked to better life quality and lower risk of death (Nagata et al., 2002; Myung et al., 2009; Wu et al., 2012; Zhang et al., 2015; Lu et al., 2017; Sunagawa et al., 2018; Woo et al., 2018; Katagiri et al., 2020; Abe et al., 2022). In particular, the consumption of nattō is related to a significant prevention of cardiovascular disease (Zhang et al., 2003; Kokubo et al., 2007; Nagata et al., 2017; Yan et al., 2017; Katagiri et al., 2020; Rodriguez Ayala et al., 2021; Abe et al., 2022). We envision that the bacterium *B. subtilis* present in nattō plays a key role in the human beneficial effects of this fermented food (Nagata et al., 2017; Woo et al., 2018; Katagiri et al., 2020). Here, we show that the key human-beneficial component of nattō is the bacterium *B. subtilis* var. natto (i.e., *B. subtilis* DG101).

## Materials and methods

### Strains source and growth media

To isolate *B. subtilis* from nattō, 250 g of nattō were heated at 80°C for 20 min to inactivate (kill) vegetative bacteria and leave only spores as viable cells (Lombardía et al., 2006). Aliquots from serial dilutions of heat-treated nattō were seeded on Luria-Bertani agar (LBA) plates and incubated for 36 h at 37°C. This procedure was repeated three times independently and in all the repetitions only one type of heat-resistant bacterial isolate was obtained. These nattō isolates and the laboratory *B. subtilis* 168 strains were cultured on Luria-Bertani (LB) broth except when another broth medium is indicated (Pedrido et al., 2013). The growth media for the other bacteria and fungi used in this work were: trypticase soy broth (TSB) for *Staphylococcus aureus*; brain heart infusion (BHI) broth for *Enterococcus faecalis* and *Listeria monocytogenes*; Chapman broth for MR *S. aureus*; MacConkey broth for *Salmonella enterica*, *Salmonella typhimurium,* and *Klebsiella pneumoniae*; MacConkey Agar with Sorbitol for EHEC O157:H7; thiosulfate-citrate-bile salts-sucrose (TCBS) broth for *Vibrio cholerae*; King A broth for *Pseudomonas aeruginosa*; and potato dextrose agar (PDA) for fungi growth. Schaeffer’s sporulation medium (SM) was used to obtain *B. subtilis* spores (Pedrido et al., 2013). The efficiency of spore formation was measured by counting viable vegetative and spore cells before and after heat treatment at 80°C, respectively, as previously described (Pedrido et al., 2013). To prepare cell suspensions, *B. subtilis* (168 and DG101 strains) and *Escherichia coli* DH5α (used as negative control) were grown in SM or LB broth, respectively, at 37°C with shaking (7,258 × g) during 24 h. The *B. subtilis* cultures were heated at 80°C for 20 min to kill cells that did not form spores. Then, the bacterial cultures (i.e., *E. coli* and *B. subtilis*) were centrifuged (3,578 × g, 15 min at 4°C) and the pellets were brought to a final concentration of 5 × 10^8^ colony forming units per milliliter (CFU/mL) in tris buffer solution (10 mM; pH 7.0) and used immediately.

### Molecular characterization of *Bacillus subtilis* DG101 isolated from nattō food

The presumptive *B. subtilis* natto isolate was microbiologically characterized using routine nutritional and biochemical tests (see Results section). The molecular typification was performed by the Sequencing Service, Molecular Biology Department of The Pasteur Institute of Montevideo, Uruguay.^1^ Briefly, a sample of chromosomal DNA from the presumptive *B. subtilis* natto isolate was used for PCR amplification using consensus primers for the 16S ribosomal RNA gene from the *B. subtilis* group. The sequences of the used forward and reverse primers were 5′-AGAGTTTGATCMTGGCTC-3′ and 5′-ATACTAGCGACTCCGACTTC-3′, respectively. The amplification product (~1,500 bp) was purified, sequenced, and compared with the sequences deposited in GenBank using the BLAST tool^2^ and in the Ribosomal Database Project.^3^ A phylogenetic tree was constructed based on *16S rDNA* sequences retrieved from the GenBank database of bacilli and LAB (i.e., *Lactobacillus* and *Bifidobacterium*). The best match of the isolate from nattō was with the 16S ribosomal coding gene of the *B. subtilis* natto BEST195 strain. The 1,407 bp DNA sequence corresponding to the 16S ribosomal gene of the new nattō isolate was deposited in GenBank under the accession number OQ813498.^4^

### Resistance to lysozyme degradation

Five mL aliquots of *B. subtilis* DG101 or *E. coli* DH5α (5 × 10^8^ CFU/mL) were independently incubated in the presence of 0, 50, 100, 150, 200, and 250 μ/mL of lysozyme at 37°C. After 20 min (Zheng et al., 1988; Kuwana et al., 2005), an aliquot of each bacterial suspension was taken, and serial dilutions were made in 0.1 M phosphate-buffered saline (PBS) pH 6.2. The corresponding dilutions were seeded with a Drygalsky spatula in LB agar (LBA) plates and after 24 h of incubation at 37°C, the viable cells (CFU) were counted.

### Acid tolerance test

To determine the pH resistance of the studied bacteria (*B. subtilis* DG101 and *E. coli* DH5α), 10 mL of each bacterial suspension were centrifuged at 3,578 × g for 10 min at 4°C and the cell pellet was reconstituted in the same original volume of sterile distilled water (5 × 10^8^ CFU/mL). The suspensions were brought to different pH (i.e., 1.5, 2.5, and 3.5) with 0.1 M HCl. The bacterial samples were incubated at 37°C for 2 h, considering this is an estimated/average physiological time of gastric emptying with a pH range of 1.5–3.5 (Hellmig et al., 2006; Beasley et al., 2015). The control pH test was carried out at pH 6.2 in 0.1 M PBS. Following the incubation times, serial dilutions of the cells were made in 0.1 M PBS (pH 6.2) to neutralize the acidity of the medium (when necessary), and different aliquots were seeded with a Drygalsky spatula in LBA plates. After 24 h of incubation at 37°C, the viable cells (CFU) were counted. The survival rate was calculated as the percentage of CFU growing in LBA compared to the initial concentration of cells, before exposition to the different pH (Hyronimus et al., 2000).

### Temperature and salt tolerance test

Briefly, the temperature and salt tolerance assays were performed in 50 mL bottles filled with 10 mL of LB broth supplemented with NaCl at 0.5, 2, 5, 7, and 10%, and *B. subtilis* DG101 (5 × 10^5^ CFU/mL). Samples were grown at 30, 37, 42, 50, and 54°C with shaking (7.258 × g) for 24 h. After the overnight growth, different dilutions of the salt and temperature-treated cultures were seeded with a Drygalsky spatula in LBA plates and after 24 h of incubation at 37°C, the viable cells (CFU) were counted. The culture that grew in unmodified LB (i.e., LB containing 0.5% NaCl) at 30°C was considered as the control and the final growth yield of the remaining cultures was expressed as a % of the growth yield of the control culture (7.5 × 10^8^ CFU/mL).

### Bile salt tolerance test

Five mL of *B. subtilis* DG101 or *E. coli* DH5α suspensions (5 × 10^8^ CFU/mL) were centrifuged at 3,578 × g for 10 min and each pellet was re-suspended in 10 mL of 0.3% bile salt solution (50% sodium cholate and 50% sodium deoxycholate) or in sterile distilled water as a control (Duc et al., 2003). The prepared suspensions were incubated at 37°C for 0, 1, 2, 3, and 4 h. Next, aliquots were taken, and serial dilutions were made in 0.1 M PBS pH 6.2. The corresponding dilutions were seeded with a Drygalsky spatula in LBA plates and after 24 h of incubation at 37°C, the viable cells (CFU) were counted. The survival rate was calculated as the percentage of CFU obtained in LBA compared to the initial cell concentration. The InfoStat 2016 and Sigmaplot 10.0 programs were used to perform the graphs.

### Production of exoenzymes

*B. subtilis* DG101 was screened for the production of exoenzymes related to the digestion of nutrients of different natures. Briefly, *B. subtilis* DG101 was streaked on LBA plates containing one of the following nutrient substrates: gelatin (LBA-gelatin 10 g/L as indicator of protease activity), pectin (LBA-pectine 5 g/L for peptinolytic activity), carboxymethylcellulose (LBA-CMC 10 g/L for cellulolytic activity), Tween-80 (LBA-Tween 80 15 mL/L for lipolytic activity), pythate (LBA-phytate 5 g/L for hydrolytic activity related to release of soluble phosphate), and starch (LBA-soluble starch 10 g/L for amylase activity). The Petri dishes streaked with *B. subtilis* DG101 were incubated at 37°C for 36 h, and the development of a clear zone around the colonies was indicative of the presence of exoenzyme activity (Lee et al., 2005; Hossain et al., 2020). For the determination of hemolytic activity, suspensions of *B. subtilis* DG101 were streaked onto blood agar plates (5%–10% sheep blood per plate) and incubated for 24 h at 37°C. The isolates were then examined for the presence of clear zones surrounding the colonies. Clear zones are considered as beta hemolysis, greenish zones as alpha hemolysis, and the absence of hemolysis zones is known as gamma hemolysis (Gerhardt, 1994).

### Metal bioremediation assays

*B. subtilis* DG101 was incubated in LB broth supplemented with the corresponding metal: cadmium (Cd_(II)_), chromium (Cr_(VI)_), or arsenite (As_(III)_) at 0, 10, 50, 100, 150, 200, 250, and 300 ppm at 37°C with shaking (5 × g). After 20 h, the bacterial cultures were centrifuged and the determination of metal concentration was performed on the cell-free supernatants (i.e., remaining metal in the supernatant). The concentration of Cr_(VI)_ was determined by a colorimetric reaction based on the formation of a complex between Cr_(VI)_ and 2,4-difenylcarbazide in an acid solution to a *λ* = 540 nm, as previously described (Megharaj et al., 2003). The concentration of As_(V)_ was determined by a colorimetric reaction based on the formation of a red complex, between arsenic hydride with silver diethyl-carbamate, to a *λ* = 530 nm (Upadhyay et al., 2018). Total chromium (Cr_(VI)_ plus Cr_(III)_), Cd_(II)_, and As (As_(V)_ plus As_(III)_) were determined by atomic absorption spectrophotometric determination at *λ* = 357.3 nm, *λ* = 228.8 nm, and *λ* = 193.7 nm, respectively (Nakahara et al., 1973; Megharaj et al., 2003; Liu et al., 2006; Cheng and Li, 2009; Guibaud et al., 2009; Singh and Singh, 2014; Upadhyay et al., 2018).

### Antimicrobial activity against pathogens

The selected methodology to test for antimicrobial activity of *B. subtilis* DG101 against microbial pathogens was adapted from the agar diffusion method (Bauer et al., 1959, 1966; Bonev et al., 2008). Briefly, the antimicrobial activity of *B. subtilis* DG101 against pathogenic bacteria and fungi (*Candida albicans*, *Fusarium verticillioides*, *Fusarium graminearum*, *Aspergillus flavus*, *Penicillium* sp.) was performed on nutrient agar (NA) or PDA plates, respectively. Then, 100 μL of overnight cultures of the different microbial pathogens to be tested were individually poured, using a Drygalsky spatula, on the corresponding type of agar plate (NA or PDA plates). After the seeded microorganisms were totally absorbed in the medium, three sterile paper disks (4 mm in diameter) pre-absorbed with 15 μL of an overnight culture of *B. subtilis* DG101 grew on LB broth (~2 ×1 0^6^ CFU/paper disk) were loaded equidistantly from each other on each type of agar plate. The NA and PDA plates containing the bacterial and fungi pathogens, respectively, with the *B. subtilis* DG101-poured paper disks were incubated for 24 h at 37°C or 72–96 h at 30°C, respectively. The clear zone formed around the paper disks indicated the growth inhibition of the tested pathogen. The inhibition halo of each bacterial pathogen produced by *B. subtilis* DG101 was measured and used to classify the pathogen as sensitive or resistant based on the recommendations of the American Society for Microbiology for antimicrobial control.^5^ For the determination of susceptibility of fungal pathogens to *B. subtilis* DG101, the fungal growth inhibition index was calculated from measurements of fungal radial growth toward (X1) versus perpendicular to (X2) the bacterial colony according to the formula [1 − (X1/X2)] *100 (Quecine et al., 2015; Bartolini et al., 2019). Paper disks pre-absorbed with sterile water were used as negative controls of antimicrobial activity.

### Assessment of antibiotic resistance of *Bacillus subtilis* DG101

The antibiotic sensitivity of *B. subtilis* DG101 was tested against the following antibiotics commonly used in hospitals: amikacin, ampicillin/sulbactam, cephalothin, cefepime, cefoxitin, ceftazidime, cilastatin, ciprofloxacin, erythromycin, gentamicin, imipenem, nitrofurantoin, norfloxacin, oxacillin, penicillin, piperacillin/tazobactam, tetracycline, trimethoprim/sulfamethoxazole and vancomycin. All the selected antibiotics are on the lists of recommended antibiotics by the U.S. Food and Drug Administration (FDA), the Center for Drug Evaluation and Research (CDER), the Clinical and Laboratory Standards Institute (CLSI), and The European Committee on Antimicrobial Susceptibility Testing (EUCAST). Briefly, commercial disks containing an appropriate amount of each antibiotic were added to Mueller Hinton agar plates, previously poured with *B. subtilis* DG101, and incubated under aerobic conditions at 37°C for 24–48 h. The inhibition zones (clear zone, in mm) were measured using a caliper. All experiments were performed in triplicate on the same day. The breakpoint values used for categorizing the microorganisms as resistant were according to the Clinical and Laboratory Standards Institute (2023).

### Biofilm experiments

For biofilm formation, *B. subtilis* DG101 and *B. subtilis* 168 cultures were grown in LB medium until they reached the stationary phase. Then, 50 μL of each culture was diluted in 2 mL of fresh LBY medium (LB broth supplemented with 4.0% yeast extract; Pedrido et al., 2013) and statically grown at 37°C for 36 h. For quantification of formed biofilm (biofilm mass), we measured the biofilm mass that adhered to the surface of the microtiter plate wells (Pedrido et al., 2013). To this end, *B. subtilis* (DG101 or 168) cells were grown in 96-well polyvinylchloride (PVC) microtiter plates at 37°C for 36 h in LBY medium. The bacterial inoculum for the microtiter plates was obtained by growing the cells in LB to mid-exponential phase and then diluting the cells to an OD_600 nm_ of 0.08 in LBY and biofilm formation was monitored by staining with crystal violet (CV) as described by Pedrido et al. (2013). Growth medium and non-adherent cells were carefully removed from the microtiter plate wells. The microtiter plate wells were incubated at 60°C for 30 min. Biofilms were stained with 300 μL of 0.3% CV in 30% methanol at room temperature for 30 min. Excess CV was removed, and the wells were rinsed with water. The CV from stained biofilms was then solubilized in 200 μL of 80% ethanol/20% acetone. Biofilm formation was quantified by measuring the absorbance at 595 nm for each well using a Beckman Coulter Multimode Detector (DTX 880).

### Adherence assays of *Bacillus subtilis* to extracellular matrix proteins

Briefly, microtiter plates were coated with 100 μL of each solution containing the following extracellular matrix (ECM) proteins (Sigma-Aldrich): fibronectin, collagen, and gelatine (10 μg/mL in bicarbonate buffer pH 9.6); and subsequently incubated overnight at 4°C. The unfixed proteins were removed, and plates were washed three times with PBS pH 8. After blocking with 0.5% non-fat milk (in PBS), to prevent non-specific bacterial binding, wells were washed again twice with PBS. Finally, bacterial suspensions of *B. subtilis* DG101 or *B. subtilis* 168 (100 μL, 1×10^8^ CFU/mL) were added and the plates were incubated on an orbital shaker for 2 h at 37°C. Unbound bacteria were removed by washing the wells five times with PBS. Adhered bacteria to the wells were fixed at 60°C for 20 min and stained with 100 μL/well of CV (0.3% in methanol—H_2_O 30% v/v) for 45 min as previously described (Styriak et al., 1999; Rodriguez Ayala et al., 2017a). The wells were subsequently washed extensively with PBS to remove the unbound stain. After adding 100 μL of citrate buffer pH 4.3 to each well and 45 min incubation at room temperature (to release the stain bound to bacteria), the absorbance values (A_595 nm_) were determined in a Multiscan enzyme-linked immunosorbent assay reader as described (Sun et al., 2019). The average values of three independent experiments performed in quadruplicate were calculated. The specificity of binding was tested using BSA as the immobilized protein. Data are expressed as the mean absorbance value (A_570 nm_), with non-specific binding corrected by subtraction.

### *In vivo* gut colonization

**Figure 1:**
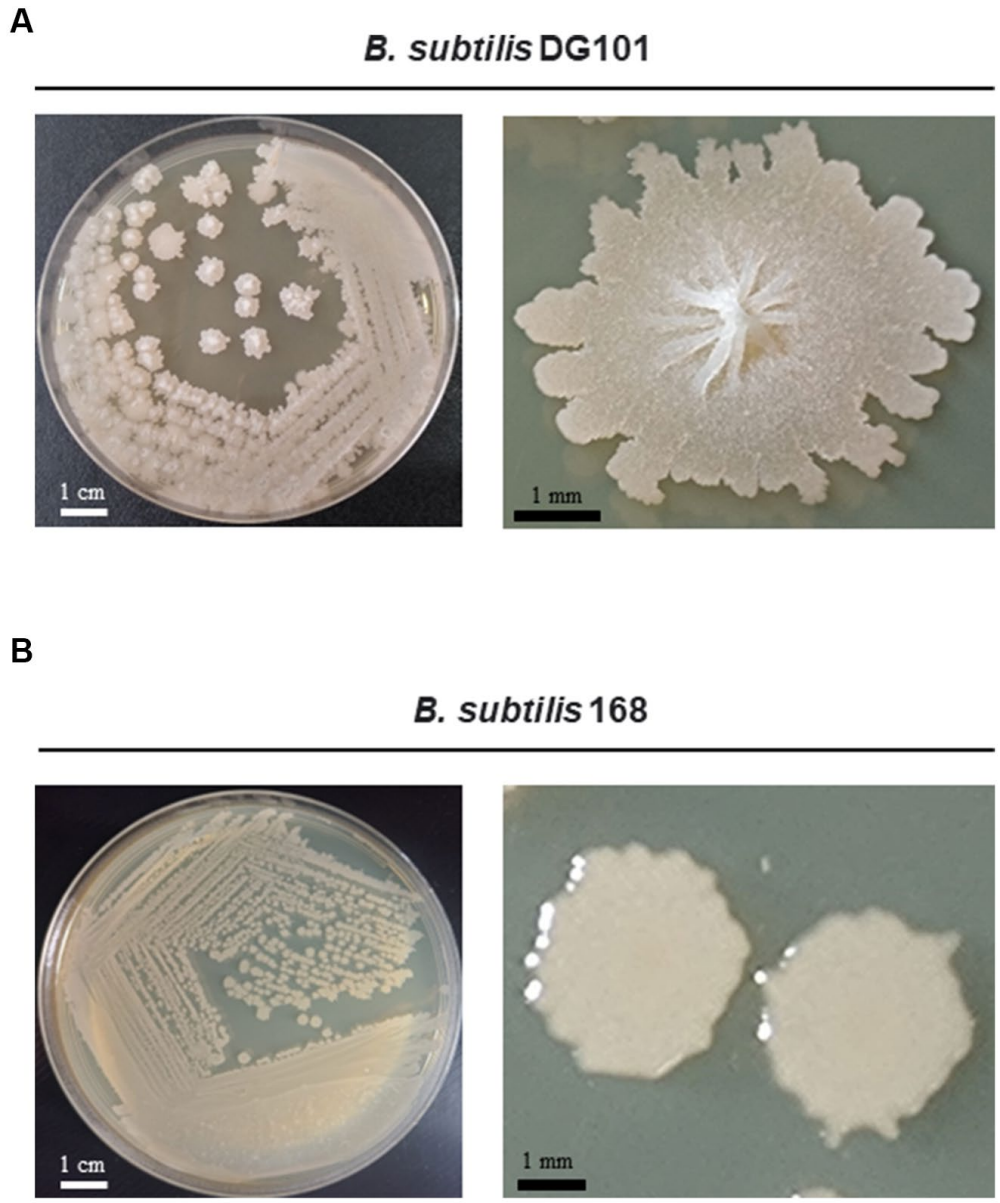
Growth of *Bacillus subtilis* DG101 on LB agar media. Morphological appearance of *B. subtilis* DG101 isolated from nattō food after incubation for 24 h at 37°C on LB agar **(A)** compared to the domesticated and non-probiotic strain *B. subtilis* 168 **(B)**. Shown are the scale bars.

The colonization of the GI tract by *B. subtilis* DG101 was performed according to Hoa et al. (2001). Briefly, *B. subtilis* DG101 eliminated in feces was monitored in Sprague Dawley rats (male, 6 to 8 weeks old, *n* = 12) housed individually in cages with gridded floors. Animals were deprived of drinking water for 6 h and then fed on a single dose of 2 × 10^7^ spores of *B. subtilis* DG101 contained in a volume of 20 mL of drinking water. After this procedure, the animals were fed *ad libitum* with an unrestricted drinking water supply. Total feces were collected day to day and weighed for 2 weeks. To determine the total number of *B. subtilis* DG101 viable cells, including spores and vegetative cells in feces, samples were suspended in 10 to 30 mL of 0.1 M PBS with frequent and rigorous vortexing. Serial dilutions were made with PBS, seeded on LBA plates, and incubated at 37°C for 2 days. *B. subtilis* DG101 colonies were identified by their characteristic colony morphology (see Figure 1). To determine the total number of *B. subtilis* DG101 spores, the same procedure was used, except that feces were suspended in 0.1 M PBS and incubated at 80°C for 20 min to inactivate (i.e., kill) vegetative cells. The total CFU count was extrapolated for the total weight of feces collected and represented as a percentage of the initial dose.

### Lipid and protein quantifications in the serum of animals fed with *Bacillus subtilis* DG101

Three-weeks-old Wistar male rats (*n* = 14) were housed in metal cages (2 animals/cage) in a temperature-controlled room under a 12 h light/dark cycle. After a 1 week adaptation period on powder chow diet (Gepsa Feed Corporation), the animals were randomly assigned to 2 groups (*n* = 7): Control group (rats fed *ad libitum* on powder chow) and Treated group (rats fed *ad libitum* on powder chow supplemented with *B. subtilis* DG101 1 × 10^10^ CFU/kg of food). On day 120, blood samples were drawn from the tail rats. Serum was separated by centrifuging the blood at 7,500 × g for 15 min. Triglyceride (TG, Cat. 1780101), total cholesterol (TC, Cat. 1220101), high-density lipoprotein-cholesterol (HDL-c, Cat. 1220103), and low-density lipoprotein-cholesterol (LDL-c, Cat. 1220108) were measured using enzymatic reagent kits from Wiener Lab (Wiener Group Co, Argentina).

For protein quantification under normal and dietary restriction conditions, 20 male Wistar rats, 3 weeks old, were housed in metal cages (2 animals/cage) in a temperature-controlled room under a 12 h light/dark cycle. After a 1 week adaptation period on a powder chow diet, the rats were randomly assigned to 4 groups (*n* = 5): Control group (rats fed *ad libitum*, consuming an average amount of 26 g of food/day/animal), Treated group (rats fed *ad libitum* and supplemented with *B. subtilis* DG101 1 × 10^10^ spores/kg of food), Nutritional stress group (rats with a dietary restriction that consisted of ~75% of the average amount of food consumed by day −19.5 g of food/day/animal-under physiological conditions), and Treated nutritional stress group (rats with dietary restriction supplemented with *B. subtilis* DG101 1 × 10^10^ spores/kg of food). After 90 days, blood samples were drawn from the tail rats. Serum was separated by centrifuging the blood at 7,500 × g for 15 min. Total proteins (TP, Cat. 1999736), albumin (ALB, Cat. 1009240), and globulins (TG, Cat. 1690004) were measured using enzymatic reagent kits from Wiener Lab. All aspects of the experiment were conducted according to the guidelines provided by the ethical committee of experimental animal care at the National University of Rosario (Santa Fe, Argentina).

### Lifespan analysis

Lifespans for the wild-type *C. elegans* N2 strain (Bristol strain) were monitored at 20°C as previously described (Cogliati et al., 2020). Briefly, worm embryos were isolated by exposing hermaphrodite adult worms to alkaline hypochlorite treatment for 3 min, and synchronized eggs were allowed to develop. In all cases, L4/young adult worms (*n* = 100) were used at time zero for lifespan analysis; they were transferred to fresh nematode growth medium agar (NGMA) plates previously seeded with *E. coli* OP50 or *B. subtilis* DG101 or *B. subtilis* 168 cells each day until the death of the last worm of each assay (Cogliati et al., 2020). Worms were considered dead when they ceased pharyngeal pumping and did not respond to prodding with a platinum wire. Worms with internal hatching were removed from the plates and excluded from lifespan calculations.

### Chemotaxis analysis

*B. subtilis* 168- or *B. subtilis* DG101-fed N2 (10 days-old adults) worms were collected, washed three times with M9 buffer (Cogliati et al., 2020), and seeded in 10 cm Petri dishes prepared with NGMA without food for 1 h. Then, approximately 75 worms were placed in the center of 6 cm plates prepared with NGMA. After all the animals were transferred to the center of the assay plates, 2 μL of attractant or repellent were seeded 2 cm from the center of the plate, and 2 μL of solvent (control) in which the attractant or repellent was diluted were seeded equidistantly. Both the attractant, repellent, and solvent (control) were added with a 1-μL drop of 1 M azide. The plates were incubated for 1 h at 25°C. Then, worms found at each end of the plates were counted, and the chemotaxis index (CI) was calculated (Bargmann et al., 1993). The attractant compounds used for the assays were 0.5% diacetyl (DA, Sigma-Aldrich) diluted in ethanol and 1 mM isoamyl alcohol (IAA; Sigma-Aldrich) diluted in water, 1 mM 2,3-pentanedione and 1 mM 2-butanone (Sigma-Aldrich). The repellent compounds used for the assays were 0.3% octanol and 15% 2-nonanone (Sigma-Aldrich). The CI was defined as the number of worms at the attractant or repellent location—the number of worms at the control location divided by the total number of worms on the plate (Bargmann et al., 1993).

### Statistical analysis

All experiments were performed in triplicate and the mean values along with the standard deviation are given. All the data from laboratory experiments were analyzed separately for each experiment and were subjected to an analysis of variance (ANOVA test). Significant effects were determined by the *F* value (*p* ≤ 0.05). For *C. elegans* survival assays, experiments were performed at least three times in duplicate. Mean survival days, standard error of the mean (S.E.M.), intervals of mean survival days with 95% confidence, and equality *p*-values to compare averages were calculated by log-rank and Kaplan–Meier tests using the OASIS program. The S.E.M. values are used in the figures; *p* < 0.1 was considered statistically significant.

## Results

### Biochemical and molecular characterization of *Bacillus subtilis* isolated from nattō food

**Figure 2:**
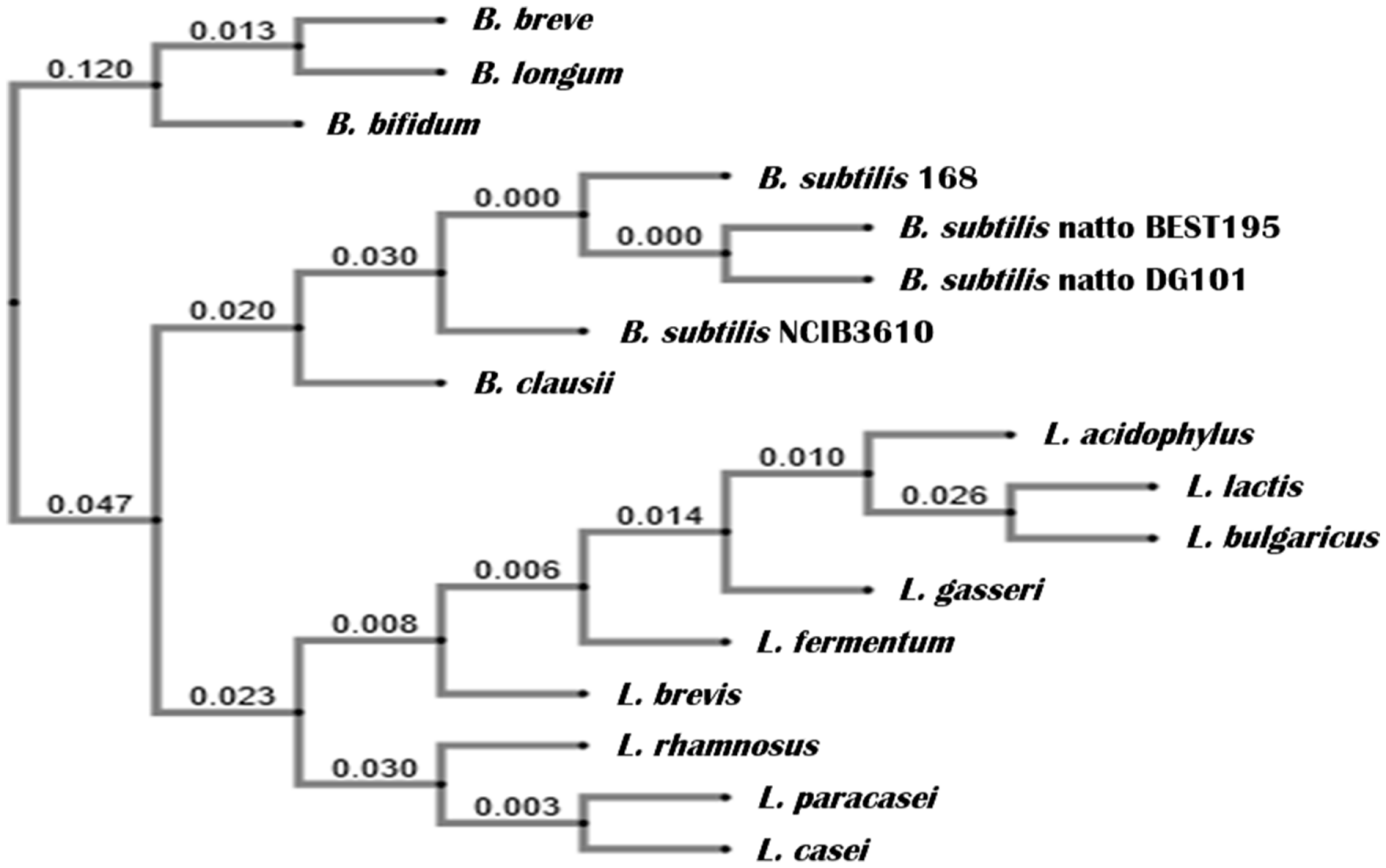
Phylogenetic relationship of the *16S rRNA* gene of *B. subtilis* DG101 within representative Bacillaceae bacteria. The genes coding for the *16S rRNA* were used to generate a neighbor-joining tree using crustal omega (www.ebi.ac.uk/tools/msa/clustalo). The phylogenetic distance with a gene encoding for *16S rRNA* of *Bifidobacterium* and *Lactobacillus* is also shown, forming two independent clusters. The numbers are Bootstrap numbers, ranging from 0 to 1, with 0 showing complete identity and no identity by 1.

The genetic pathways (e.g., the phosphorelay signaling and the Spo0A/SinR/AbrB/LuxS regulatory circuit) controlling the multicellular behavior of the *B. subtilis* natto strain described here have been previously reported (Lombardía et al., 2006; Pedrido et al., 2013; Grau et al., 2015: Rodriguez Ayala et al., 2017a). Now, we present a short discussion of the biofilm, sliding, and adherence proficiencies of the natto isolate plus a profound and complete description of its *in vitro* and *in vivo* probiotic properties. The bacterial nattō isolate (see Material and methods) showed a complex colony architecture, resembling the sophisticated colony (biofilm) architecture of wild (undomesticated) *B. subtilis* isolates (Branda et al., 2001), (Figure 1A). Accordingly, the biofilm physiognomy of the nattō isolate remarkably differed from the simple and undifferentiated colony (biofilm) architecture of domesticated *B. subtilis* isolates (Branda et al., 2001), i.e., 168 strain (Figure 1B). The confirmation that the nattō isolate belongs to *B. subtilis* was achieved through Sanger sequencing and alignment of the 16S ribosomal RNA (rRNA) encoding gene (Figure 2, see Material and methods for details). The nattō isolate exhibited strong homologies (~99.7% similarity and ~99.5% identity over its full length) to the *16S rRNA* sequences belonging to different *B. subtilis* strains, and significant homology with other Gram-positive probiotic bacteria of the genus *Lactobacilli* and *Bifidobacterium* (Figure 2). Interestingly, the *16S rRNA* gene of the nattō isolate showed the strongest homology with the BEST 195 *B. subtilis* natto strain previously characterized (Kamada et al., 2014). Therefore, the nattō isolate (Figures 1A, 2) was given initially the name *Bacillus subtilis* RG4365 (**Roberto Grau**; Lombardía et al., 2006; Pedrido et al., 2013; Grau et al., 2015; Rodriguez Ayala et al., 2017a) and more recently, because of its approval for consumption as a human probiotic, *B. subtilis* DG101 (**Doctor Grau**; Cardinali et al., 2020; Rodriguez Ayala et al., 2021, 2022a, 2022b). Both strain designations (i.e., RG4365 and DG101) refers to the same nattō strain. The 1,407-base sequence fragment of the *16S rRNA* gene of *B. subtilis* DG101 was deposited in GenBank (accession number OQ813498).

*B. subtilis* DG101 was initially characterized by Gram staining, sporulation proficiency, and basic biochemical and physiological tests whose results correlated well with the expected biochemistry and physiology attributes of bacilli (Table 1A). *B. subtilis* DG101 was proficient to grow in the presence of high salt concentrations at different growth temperatures (Table 1B) and in the production of several exoenzymes (Table 1C) involved in the digestion of diverse types of macromolecules (i.e., polysaccharides, complex lipids, proteins). The *in-situ* production of these exoenzymes in the host intestine could play a positive role in improving food diet digestion. Importantly, *B. subtilis* DG101 lacked hemolysis activity (Table 1C) and was sensitive to antibiotics routinely used to treat infections (Table 1D).

**Table 1:** Microbiological characterization of *B. subtilis* DG101.

A. Morphological and biochemical test for *B. subtilis* DG101	
Cell morphology	rods
Gram staining	Gram (+)
Schaeffer–Fulton staining	+
Catalase test	+
Oxidase test	−
Sporulation proficiency	+
Biofilm formation	+
Flagella	−
Motility in liquid	−
Motility on surface	+

Growth temperature (°C)					
NaCl (%)	30	37	42	50	54
B. Proficiency of *B. subtilis* DG101 to grow at different temperatures and salt concentrations. The *B. subtilis* DG101 growth at each tested salt concentration/growth temperature condition is indicated as the % related to the growth of a *B. subtilis* DG101 culture grew at 30°C without salt supplementation					
0.5	100 (control)	100	100	80	70
2	100	100	100	80	70
5	100	100	100	80	70
7	100	100	100	70	60
10	90	90	85	65	50

C. Extracellular enzymes produced by *B. subtilis* DG101	
Amylase	+
Cellulolytic	+
Lignilolytic	−
Phyatse	+
Lipase	+
Protease	+
Hemolysis	−

Antibiotic	Resistant ≤ (mm)	Intermediate (mm)	Sensitive ≥ (mm)	Obtained diameter	Antobiotic Susceptibility
D. Antibiotic susceptibility pattern of *B. subtilis* DG101. See Material and methods and results section for details					
Amikacin	14	15–16	17	42	Sensitive
Ampicillin/Sulbactam	11	12–14	15	40	Sensitive
Cephalothin	14	15–17	18	52	Sensitive
Cefepime	14	15–17	18	25	Sensitive
Cefoxitin	14	15–17	18	38	Sensitive
Ceftazidime	14	15–17	18	20	Sensitive
Cilastatin	14	15–17	18	20	Sensitive
Ciprofloxacin	15	16–20	21	40	Sensitive
Erytromycin	13	14–22	23	36	Sensitive
Gentamicin	12	13–14	15	36	Sensitive
Imipenem	15	16–20	21	42	Sensitive
Nitrofutantoin	14	15–16	17	26	Sensitive
Norfloxacin	12	13–16	17	29	Sensitive
Oxacillin	10	11–12	13	20	Sensitive
Penicillin	26	27–46	47	50	Sensitive
Piperracillin/Tazobactam	14	15–18	19	48	Sensitive
Tetracycline	14	15–18	19	21	Sensitive
Trimethoprim/Sulfamethoxazole	10	11–15	16	34	Sensitive
Vancomycin	14	15–16	17	31	Sensitive

**Figure 3:**
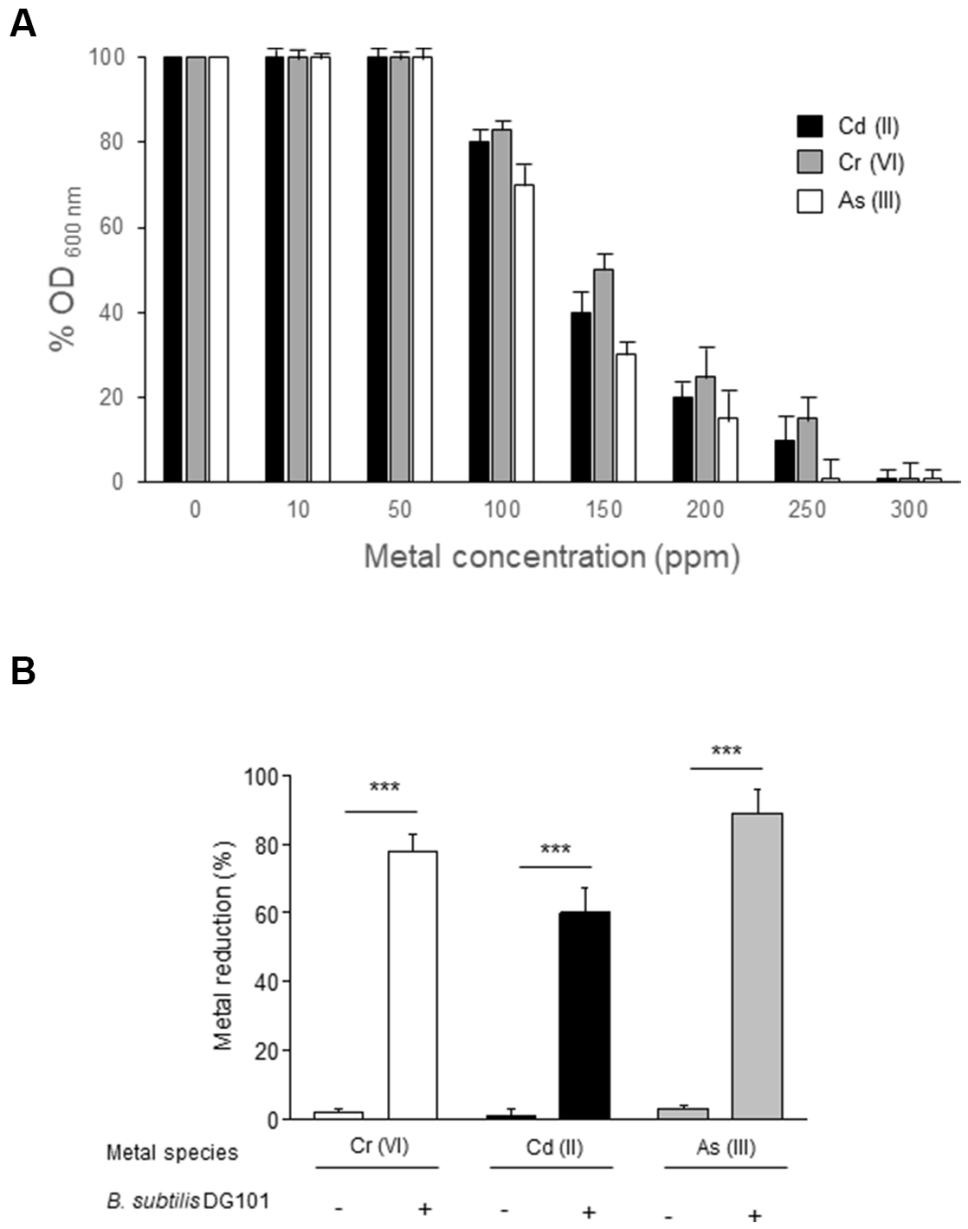
Bioremediation of toxic heavy metals Cd (II), Cr (VI), and As (III) by *B. subtilis* DG101. **(A)** Effect of different concentrations of Cd_(II)_ (black bar), Cr_(VI)_ (grey bar), and As_(III)_ (white bar) on the final yield growth of *B. subtilis* DG101 after 24 h of cultivation in LB broth, with or without metal supplementation. **(B)** Percentage of metal detoxification after 24 h of *B. subtilis* DG101 grown in LB broth supplemented with 50 ppm de each metal (Cr_(VI)_, Cd_(II)_ or As_(III)_). See Material and methods for details.

*B. subtilis* DG101 was able to grow in the presence of high concentrations of toxic metals (i.e., Cd _(II)_, Cr_(VI)_, or As_(III)_) (Figure 3). The minimal inhibitory concentrations (MICs) of *B. subtilis* DG101 growth in the presence of each metal were ~ 300 ppm for Cd _(II)_ and Cr_(VI)_; and ~250 ppm for As_(III)_ (Figure 3A). These MIC values were significantly higher than the metal limits allowed to be present in drinking water: 5 ppb, 5 ppm, and 10 ppb for Cd _(II)_, Cr_(VI)_ or As_(III)_, respectively (Nakahara et al., 1973; Singh and Singh, 2002; Megharaj et al., 2003; Liu et al., 2006; Cheng and Li, 2009; Guibaud et al., 2009; Upadhyay et al., 2018). The proficiency of *B. subtilis* DG101 to grow satisfactorily in the presence of 50 ppm of each metal (Figure 3A) correlated with the significant removal of Cd _(II)_, Cr_(VI)_, and As_(III)_ from the liquid phase of the culture (i.e., cell-free supernatant) (Figure 3B). The presence of Cd _(II)_, Cr_(VI)_, and As_(III)_ in the cell-free supernatant of *B. subtilis* DG101 that grew overnight in the presence of 50 ppm of each metal was reduced more than ~80%, ~ 60%, and ~85%, respectively (Figure 3B). The growth of *B. subtilis* DG101 in the presence of each metal correlated with the absorption of Cd _(II)_ to the cell envelope, and the reduction or oxidation of Cr_(VI)_ to Cr_(III)_ and As_(III)_ to As_(V)_, respectively (data not shown). The proficiency of *B. subtilis* DG101 to transform toxic metals into less hazardous species in humans (i.e., conversion of Cr_(VI)_ to Cr_(III)_ and As_(III)_ to As_(V)_) or a less available form (absorption of Cd_(II)_ to the cell envelope) opens the potential use of this probiotic bacilli for environmental bioremediation uses and/or prevention/treatment of food/water metal poisoning.

### Growth characterization of *Bacillus subtilis* DG101

**Figure 4:**
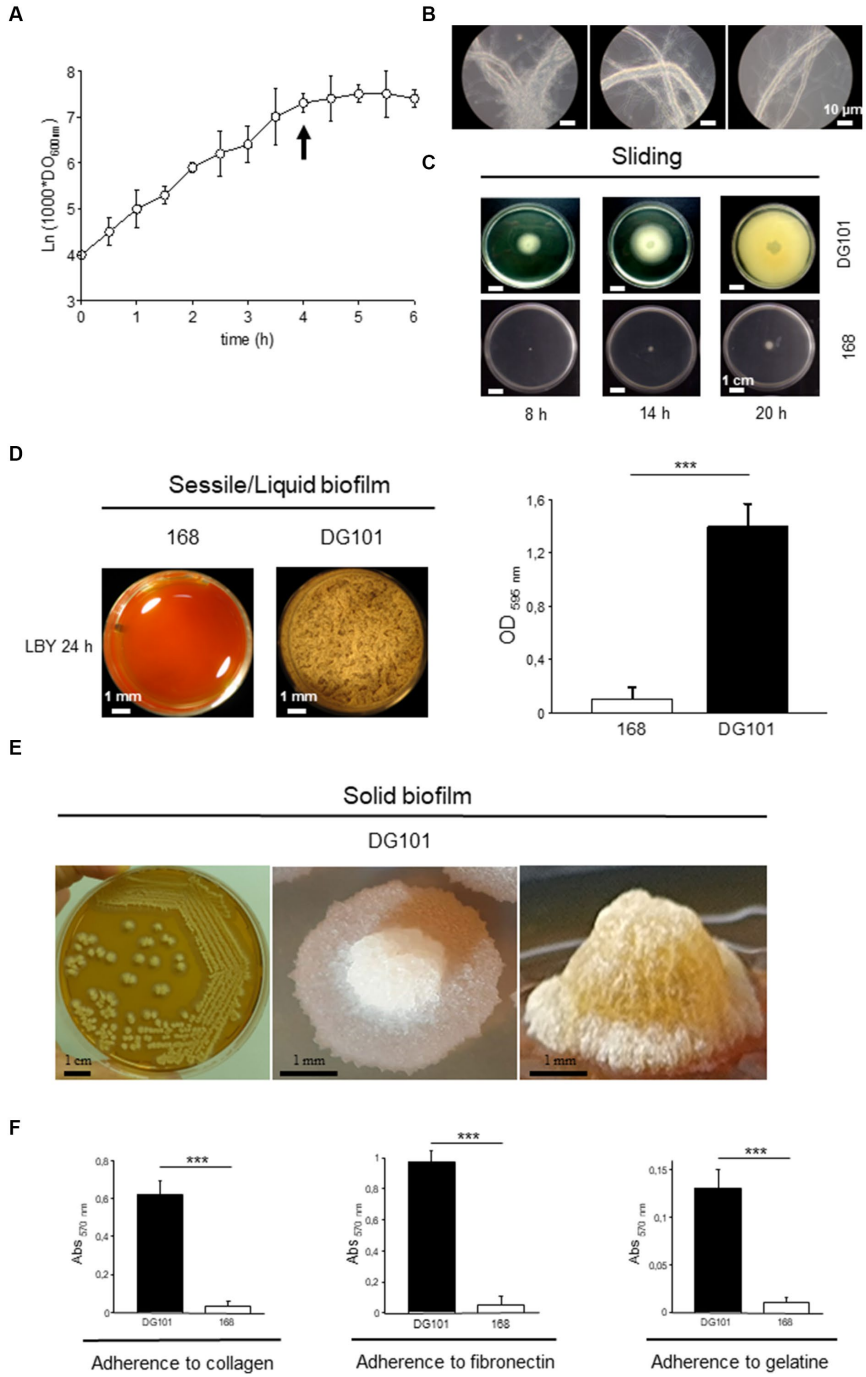
Growth behavior of *B. subtilis* DG101 in liquid and in solid media at 37°C. **(A)** Growth curve of *B. subtilis* DG101 in LB broth with shaking. **(B)** Pictures of the *B. subtilis* DG101 cellular cords observed during the log phase of growth (pictures were taken at the time indicated by the arrow in **A**). Scale bar is 10 μm. **(C)** Surface-associated motility of *B. subtilis* DG101 on soft LB agar plates. *B. subtilis* DG101 cells were cultured and inoculated on LB-0.7% agar plates and the surface translocation of the expanding cellular swarm was photographed at different times as indicated. The control showed the inability of *B. subtilis* 168 to swarm on LB-0.7% agar plates (bottom picture). Scale bar is 1 cm. **(D)** Liquid biofilm (pellicle) development of *B. subtilis* DG101 and *B. subtilis* 168 seeded in microplates containing LBY broth. Top-view pictures were taken after 20 h of incubation without agitation. Scale bar is 1 mm. Right panel, mass quantification of the biofilms produced by *B. subtilis* 168 and *B. subtilis* DG101 after 24 h of incubation (see Material and methods for details). **(E)** Solid biofilm (colony) development of *B. subtilis* DG101 on LBY agar plates. Panoramic appearance of typical *B. subtilis* DG101 colonies (biofilms) developed after 24 h of incubation are shown. Scale bar is 1 mm. **(F)** Proficiency of *B. subtilis* DG101 and *B. subtilis* 168 to bind to representative proteins of the extracellular matrix of tissues. Immobilized collagen (left panel), fibronectin (middle panel), gelatin (right panel), or BSA (not shown) were incubated in the presence of *B. subtilis* DG101 or *B. subtilis* 168 as indicated. Each data point contains the mean ± S.E.M. of a representative experiment performed by quadruplicate. NS: no significant difference, ^***^p < 0.001 (ANOVA with Bonferroni test).

The growth rate (μ) of *B. subtilis* DG101 cultured in LB broth with shaking at 37°C was ~0.0231 min^−1^ with a generation time (*t*_g_) of 30 min (Figure 4A). This growth showed two distinctive features that distinguish *B. subtilis* DG101 from other *B. subtilis* strains previously described (e.g., strains 168 and NCIB3610; Branda et al., 2001). First, as a key difference with the swimming proficiency of the *B. subtilis* 168 and NCIB3610 strains (Branda et al., 2001), *B. subtilis* DG101 was not motile in the liquid medium and this phenotype correlated with the absence of visible flagella after sample staining with a specific dye for flagella visualization (Table 1A and data not shown). Associated with the observed non-motility phenotype in liquid broth with shaking, *B. subtilis* DG101 showed a tendency to form long strings (longer than 50 μm each) constituted of individual cells lined up one after the other (Figure 4B). The tendency to form long cords of cells was observed during the entire log phase of growth and reached the maximal expression of cords formation at the transition from T_−1_ to T_0_ (the end of the log phase of growth, Figure 4B). At this developmental time (T_0_) in LB broth, a small portion (~ 15%) of the culture initiated the sporulation program, and later (approximately 2–3 h after T_0_) the cellular cords were completely dissembled, and free vegetative cells and sporangia (similar to the ones of *B. subtilis* 168 or *B. subtilis* NCIB3610, data not shown) were released into the culture medium (data not shown). Surprisingly, *B. subtilis* DG101 was able to perform surface-associated motility similar to the flagella-dependent swarming motility exhibited by *B. subtilis* NCIB3610 (Branda et al., 2001) but by a flagella-independent mechanism of sliding motility (Grau et al., 2015; Figure 4C).

Under sessile conditions of growth (i.e., without shaking) in liquid and solid medium, *B. subtilis* DG101, but not *B. subtilis* 168, formed (liquid and solid) robust, sophisticated, and long-lasting hydrophobic biofilms (Branda et al., 2004; Pedrido et al., 2013; Grau et al., 2015; and Figures 4D,E). The proficiency in biofilm formation depended on the adherence ability of the bacterium to different substrates (Pedrido et al., 2013). For example, *B. subtilis* DG101 showed a strong and specific adherence to diverse proteins present in the extracellular matrix of the human intestinal mucosa (e.g., collagen and fibronectin), an adhesive property that would be important for the competitive exclusion of pathogen adherence (VidyaLaxme et al., 2014; Rodriguez Ayala et al., 2017a; and Figure 4F).

### Antimicrobial characterization of *Bacillus subtilis* DG101

The antimicrobial activity of *B. subtilis* DG101 was assessed by measuring the growth inhibition zone diameter of different pathogenic bacteria and fungi that grew in the presence of the probiotic (see Material and methods for details). The results shown in Table 2 indicate the effectiveness of *B. subtilis* DG101 in controlling the growth of pathogenic Gram-positive and Gram-negative bacteria (Table 2A) as well as the proficiency of the probiotic to inhibit the growth of yeasts and mycotoxin-producer filamentous fungi (Table 2B).

**Table 2:** Antimicrobial activity of *B. subtilis* DG101.

Name	Strains	Inhibition zone (mm)	Susceptibility to *B. subtilis* DG101
A. Inhibition zones (mm) of growth produced by *B. subtilis* DG101 on pathogenic Gram-positive (*S. aureus*, *L. monocytogenes*, *B. cereus*, *E. faecalis*, MRSA and *S. mutans*) and Gram-negative (enterohemorrhagic *E. coli*—EHEC-, *P. aeruginosa*, *S. enteritica*, *S. typhimurium*, *V. cholerae,* and *K. pneumoniae*) bacteria			
Gram (+)			
*S. aureus*	ATCC25923	16	Sensitive
*L. monocytogenes*	ATCC13932	15	Sensitive
*S. mutans*	ATCC35668	14	Sensitive
*B. cereus*	ATCC11778	13	Sensitive
*E. faecalis*	ATCC29212	13	Sensitive
MRSA	ATCC33591	23	Sensitive
Gram (−)			
*E. coli* (EHEC)	ATCC43895	13	Sensitive
*P. aeruginosa*	ATCC27853	14	Sensitive
*S. enteritica*	ATCC13076	13	Sensitive
*S. typhimurium*	ATCC14028	13	Sensitive
*V. cholerae*	ATCC14033	12	Sensitive
*K. pneumoniae*	ART2008133	13	Sensitive

Name	Strains	Inhibition (%)	Susceptibility to *B. subtilis* DG101
B. Inhibition of pathogenic fungi (*C. albicans*, *F. verticilloides*, *F. graminearium*, *A. flavus*, *Penicillium* sp.) growth. See Material and methods for details			
*C. albicans*	ATCC210231	>80	Sensitive
*F. verticilloides*	Ccc143-2005	>80	Sensitive
*F. graminearium*	Ccc326-2010	>80	Sensitive
*A. flavus*	HaFL303	>80	Sensitive
*Penicillium* sp.	HPS1911	>80	Sensitive

### *In vitro* resistance of *Bacillus subtilis* DG101 to gastrointestinal-related conditions and *in vivo* gut colonization

**Figure 5:**
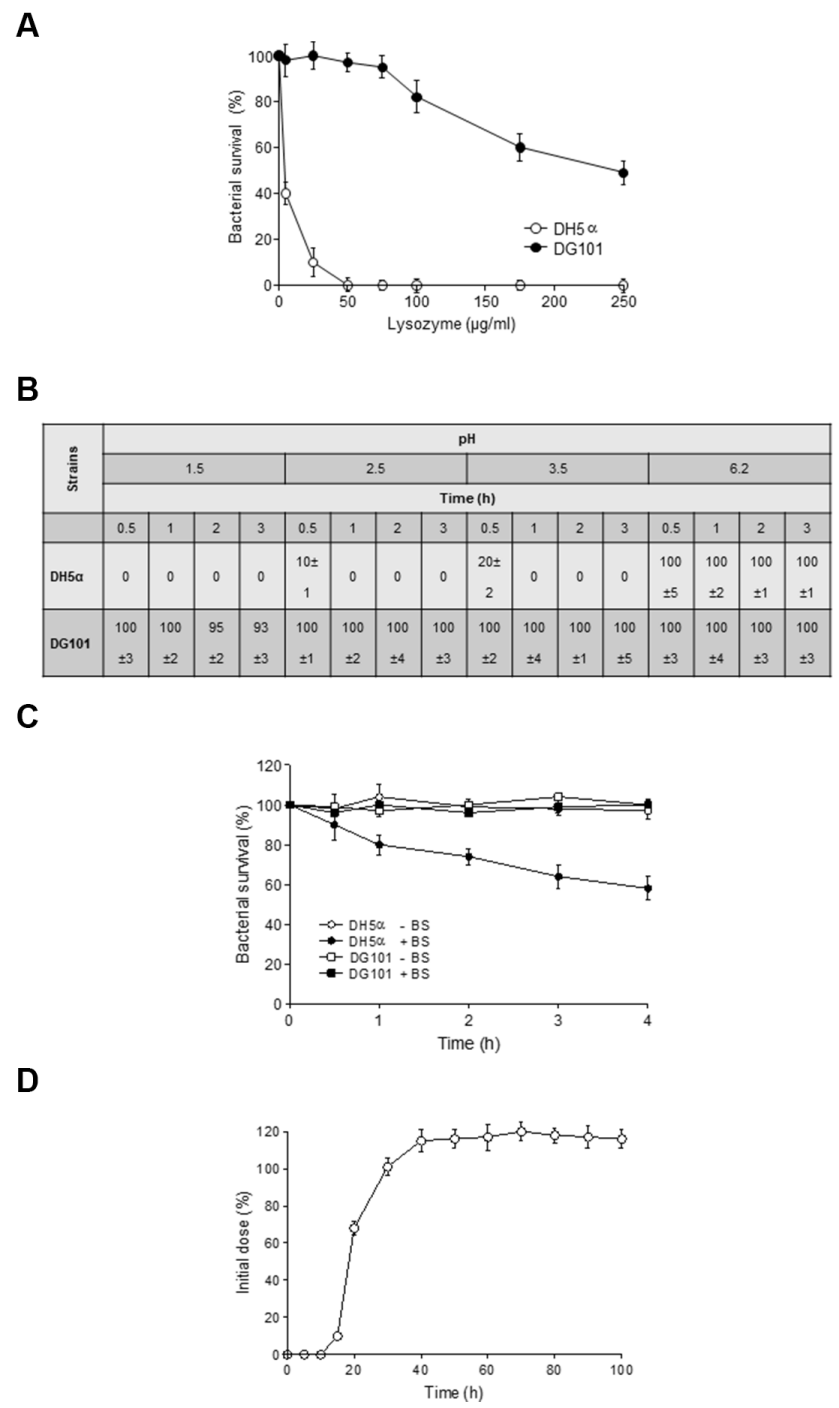
Survival of *B. subtilis* DG101 to stressful conditions of the gastrointestinal tract. **(A)** Effect of lysozyme. *E. coli* DH5α (white circles) and *B. subtilis* DG101 (black circles) suspensions (5 × 10^8^ CFU/mL) in PBS buffer were incubated in the presence of different lysozyme concentrations at 37°C for 20 min. After 20 min, the survival % for each strain was calculated. **(B)** Effect of pH. *E. coli* DH5α and *B. subtilis* DG101 (5 × 10^8^ CFU/mL) were exposed to different pH before to determine the survival expresses as %. **(C)** Effect of bile salts. *B. subtilis* DG101 and *E. coli* DH5α suspensions (5 × 10^8^ CFU/mL) were incubated in PBS buffer with and without (black and with circles, respectively) 0.3% bile salts (BS) and the survival expresses as % was determined at different times. **(D)** *In vivo* gut colonization. Persistence of *B. subtilis* DG101 in the feces of male Sprague Dawley rats fed with a single dose of *B. subtilis* DG101 at time zero. For **(A–D)**, the average % values of three independent repetitions ± S.E.M. (*n* = 3) are shown. See Material and methods for details.

A probiotic bacterium must be able to remain alive in the hostile environment of the host GI tract (Cutting, 2011). To this end, the probiotic must resist the lytic activity of the lysozyme during its transit through the buccal cavity and the low pH and bile salts present in the stomach and intestine, respectively (Zheng et al., 1988; Duc et al., 2003; Kuwana et al., 2005; De Vecchi and Drago, 2006; Hellmig et al., 2006; Beasley et al., 2015). As shown in Figure 5A, *B. subtilis* DG101 spores were resistant to the lysozyme’s hydrolysis at similar concentrations present or secreted in the human buccal cavity (Figure 5A), the low pH that resembled the exposure and transit through the stomach acidity (Figure 5B), and bile salts concentrations that correlated with physiological bile concentrations present in the human GI tract (Figure 5C).

The *in vitro* stress resistance of *B. subtilis* DG101 to GI-simulated conditions (Figures 5A–C) correlated well with its proficiency in *in vivo* gut colonization in animal models (Figure 5D). In this assay, 60 days-old Sprague Dawley male rats were fed with a single dose of 2 × 10^7^ spores of *B. subtilis* DG101 present in drinking water (20 mL), and the fecal elimination of the probiotic strain was quantified day to day for 2 weeks (see Material and methods for details). *B. subtilis* DG101 started to be present in the feces of inoculated animals approximately 15 h after the feeding, and after ~40 h, the CFU of *B. subtilis* DG101 in animal feces passed the initial inoculum of the probiotic (Figure 5D), indicating that the ingested spores were able to germinate and grow (data not shown). After 2 weeks, the total CFU present in the feces of *B. subtilis* DG101-fed animals (a total of 3.0 × 10^8^ CFU, 10% vegetative cells, and 90% spores, data not shown) exceeded by ~1.5 log the total CFU of *B. subtilis* DG101 ingested with the drinking water (2.0 × 10^7^ CFU). Interestingly, the *B. subtilis* DG101-fed animals continued with the fecal elimination of the probiotic (spores and vegetative cells, data not shown) at a rate of ~1 × 10^3^ to 5 × 10^3^ CFU/gram of fecal matter during 3 months after the end of the experiment (the CFU of spore-forming indigenous bacilli different to *B. subtilis* DG101 in rat feces was around 1 × 10^1^ to 1 × 10^2^ CFU/gram of fecal matter, data not shown). Overall, these results show that *B. subtilis* DG101 is able to persist, grow, and colonize the GI tract.

### *Bacillus subtilis* DG101 contributes to lipid and protein serum homeostasis in mammalian model

**Figure 6:**
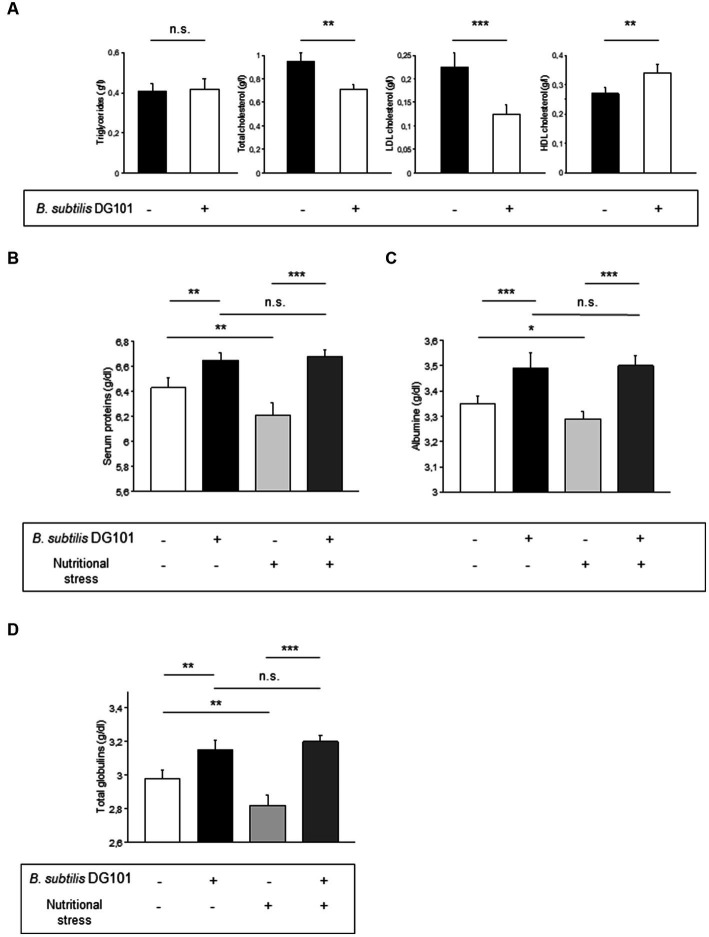
Beneficial effect of *B. subtilis* DG101 on lipid and protein serum homeostasis in an animal model. **(A)** *B. subtilis* DG101 significantly decreases total cholesterol and LDL-cholesterol levels and increases HDL-cholesterol levels in the serum of male rats (Sprague Dawley) fed with *B. subtilis* DG101 supplementation. **(B-D)** *B. subtilis* DG101 improves serum protein levels in animals under physiological and nutritional stress (dietary restriction) conditions. Figures show the level of total protein **(B)**, albumin **(C)**, and globulins **(D)**, present in the sera of 120 days-old Wistar rats fed with or without *B. subtilis* DG101 supplementation, see Material and methods for details. The bars represent the average of 2 determinations carried out each in duplicate ± S.E.M. (*n* = 5 rats per group). Asterisks indicate statistical significance (^**^p < 0.01 ^***^p < 0.001; ns, no significant difference, *p* > 0.5).

To analyze the potential beneficial effects of *B. subtilis* DG101 on blood biochemical parameters, the food of Sprague Dawley male rats, from day 1 after weaning, was supplemented with a daily dose of 1 × 10^10^ CFU of *B. subtilis* DG101/kg food (treated group). After 120 days, blood samples were taken from the animal tails to analyze and compare the lipid levels present in the serum with the lipid levels present in animals of similar age fed without the probiotic supplementation (control group). The analysis included the determination of triglycerides, total cholesterol, HDL cholesterol, and LDL cholesterol. As shown in Figure 6A, the animals of the treated group (compared with the control group animals) did not modify their triglyceride content although they showed a significant reduction of the levels of total cholesterol and LDL cholesterol (“bad cholesterol”). Accordingly, the animals of the treated group showed higher serum levels of HDL cholesterol (“good cholesterol”) compared with the control group animals (Figure 6A). These results provide experimental evidence of the beneficial effect of *B. subtilis* DG101 on serum lipid homeostasis.

Next, it was interesting to analyze the performance of *B. subtilis* DG101-fed animals under nutritional stress conditions (dietary restriction). It is known that dietary restriction (or caloric restriction) represents nutritional stress that decreases the level of serum proteins (Alexopoulos et al., 2004). Therefore, we were intrigued to evaluate if *B. subtilis* DG101 could manage this nutritional stress (e.g., maintenance of a physiologic level of plasmatic proteins) to benefit host health. To answer this question, 30 days-old male Wistar rats were divided into four groups as indicated in the Material and methods section. As shown in Figures 6B,C, the subgroup of animals fed with the supplement of *B. subtilis* DG101 and under nutritional stress showed higher levels of total serum proteins and albumin than the respective group of animals under nutritional stress and without *B. subtilis* DG101 supplementation (*p* < 0.01). The other main protein component of the serum, apart from albumin, are the globulins which are divided into alpha-1, alpha-2, beta, and gamma globulins (Abbas et al., 2011). As shown in Figure 6D, the serum concentration of globulins of rats complemented in their diet with *B. subtilis* DG101 was significantly higher in animals with or without nutritional stress. Interestingly, there were no significant differences in the levels of total serum proteins, albumin, and globulins in *B. subtilis* DG101-fed animals without nutritional stress and *B. subtilis* DG101-fed animals under nutritional stress (Figures 6B–D). These findings suggest that hosts colonized by *B. subtilis* DG101 were well-equipped to respond to the stressful condition of nutrient limitation. Overall, these results provide experimental evidence of the beneficial effect of *B. subtilis* DG101 on the maintenance of serum protein levels in healthy individuals under physiological conditions of growth or subjected to nutritional stress.

### *Bacillus subtilis* DG101 extends the healthy life expectancy in *Caenorhabditis elegans* animal model

**Figure 7:**
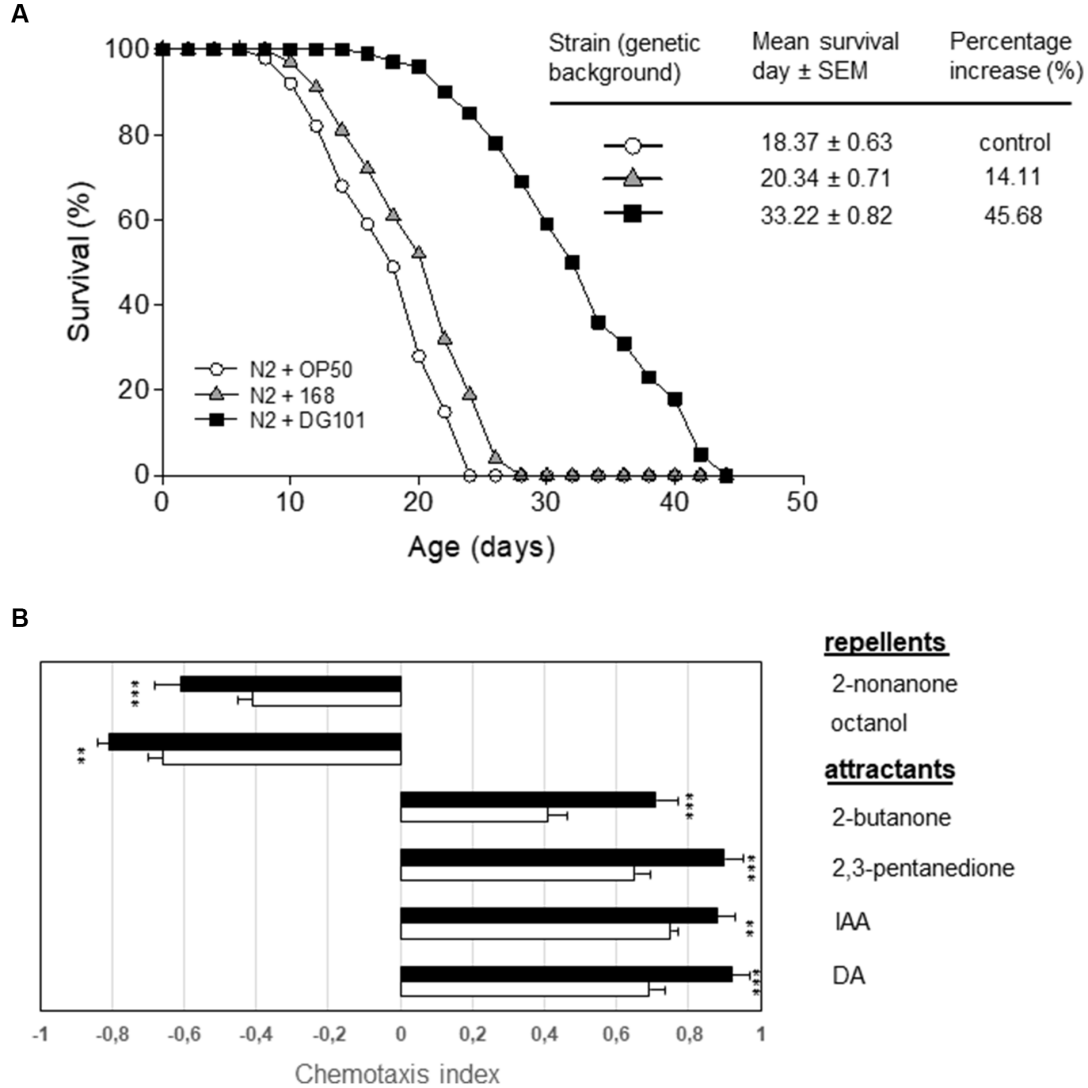
*B. subtilis* DG101 extends the *C. elegans* lifespan and improves its behavioral response. **(A)** *C. elegans* longevity fed on *B. subtilis* DG101 cells. Fifty late L4/young-adult-stage N2 worms were fed on *B. subtilis* DG101 or *B. subtilis* 168 cells (black squares and grey triangles, respectively) on NGM agar plates, and survival was monitored. The OP50 *E. coli* strain (white circles) was used as a control. The survival graph is the average ± S.E.M. (*n* = 3), and the survival increase is expressed as a percentage of the total number of worms fed on OP50 *E. coli*. **(B)** Chemotaxis indexes (CI) for N2 worms to diacetyl (DA), isoamyl alcohol (IA), 2,3-pentanedione, 2-butanone (attractants); octanol, and 2-nonanone (repellents) scored for 1 h. A typical result out of three independent experiments (performed in duplicate) is presented. Bars represent the CI response of N2 worms to each odorant ± S.E.M. Asterisks indicate statistical significance ^**^p < 0.01; ^***^p < 0.001; ns, no significant difference, *p* > 0.5.

One interesting property of some probiotic strains is the attribute to extend the host lifespan in different animal models (e.g., flies, mice, and worms), and we wondered if *B. subtilis* DG101 would possess this anti-aging property. To this end, we assayed the lifespan extension of the animal model *Caenorhabditis elegans* (Cogliati et al., 2020) when its gut was colonized by different bacterial strains including *B. subtilis* DG101. One-day-old wild-type worms of the Bristol N2 strain were grown on NGMA plates seeded with *E. coli* OP50 or *B. subtilis* DG101 (the ingested bacteria that survived the transit from the worm grinder to the gut constituted the gut flora of the animal). The NGMA plates seeded with the different bacteria were incubated at 20°C and the worm survival was monitored every day as described (see Material and methods and Donato et al., 2017). As shown in Figure 7A, N2 worms fed on *B. subtilis* DG101 gained ~45% in lifespan extension (mean survival 33.22 ± 0.82 days) when compared with the lifespan extension of the N2 worms colonized by the lab routine and the non-probiotic strain *E. coli* OP50 used to feed *C. elegans* (mean survival 18.37 ± 0.63 days, *p* < 0.001). Figure 7A also shows the lifespan extension of N2 worms fed the domesticated and non-probiotic *B. subtilis* 168 (mean survival 20.34 ± 0.71 days) compared with the lifespan of N2 worms fed *E. coli* OP50 (*p* < 0.001). Importantly, *B. subtilis* 168 and *E. coli* OP50 were much less effective than *B. subtilis* DG101 in prolonging the life expectancy of *C. elegans* when colonizing its gut.

In addition to an extension of the host life expectancy, a healthy life extension (i.e., a host aging accompanied by a delay in the behavioral decay associated with the age progression) is also desirable (Cogliati et al., 2020). One sensory behavior we examined was chemotaxis. The age-synchronized wild-type control strain (N2) of *C. elegans* was cultured at 20°C from egg hatching, using *B. subtilis* 168 or *B. subtilis* DG101 as a food source. Then, worms of 10 days of age, were starved of food (168 or DG101) for 1 h before to compare their chemotactic response toward the attractants. As shown in Figure 7B, *B. subtilis* 168-colonized N2 worms exhibited a normal chemotactic response toward DA (CI = 0.69 ± 0.02; *n* = 75), IAA (CI = 0.75 ± 0.02; *n* = 75), 2,3-pentadione (CI = 0.65 ± 0.01; *n* = 75), and 2-butanone (CI = 0.41 ± 0.02; *n* = 75) (Figure 7B, attractants, white bars). Importantly, the colonization of *C. elegans* gut by *B. subtilis* DG101 significantly improved the behavioral responses (chemotaxis index) of this host model organism to the chemical attractants [DA (CI = 092 ± 0.01; *n* = 75), IAA (CI = 0.88 ± 0.02; *n* = 75), 2,3-pentadione (CI = 0.90 ± 0.02; *n* = 75), and 2-butanone (CI = 071 ± 0.03; *n* = 75)] (Figure 7B, attractants, black bars).

Accordingly, N2 worms fed on *B. subtilis* DG101 significantly improved the response repellent stimuli [2-nonanone (CI = −0.71 ± 0.01; *n* = 75), and octanol (CI = −0.91 ± 0.02; *n* = 75) (Figure 7B, repellents, white bars)] when compared with worms colonized by *B. subtilis* 168 2-nonanone (CI = −0.41 ± 0.02; *n* = 75) and octanol (CI = −0.66 ± 0.01; *n* = 75) (Figure 7B, repellents, black bars). Therefore, *B. subtilis* DG101 possesses the potential attribute to extend the host’s healthy lifespan.

## Discussion

Probiotic bacteria do not always produce *in vivo* beneficial effects for the host. The ability to reach and colonize (in the desired number) the GI tract is sometimes compromised (Duary et al., 2011; Botta et al., 2014; Gomathi et al., 2014). For example, LAB probiotics have shown differences in their ability to survive GI conditions depending on the source of their isolation, preservation form, or packaging (e.g., powdered, or liquid form, microencapsulated, lyophilized) (Ozawa et al., 2012). Also, the accessibility of alive probiotic bacteria to the gut, and the subsequent adhesion (i.e., adherence to extracellular matrix components of the mucosa) and colonization (e.g., favored by the proficiency in biofilm formation), are important for probiotics to colonize the host gut and produce their beneficial effects (Horie et al., 2005; VidyaLaxme et al., 2014; Volstatova et al., 2016; Rodriguez Ayala et al., 2017a).

Here, we show that *B. subtilis* DG101 (Figures 1, 2) expresses many desirable attributes of a probiotic, e.g., antibiotic susceptibility, antimicrobial activity, proficiency in biofilm formation, translocation over surfaces, and adherence to ECM proteins (Figure 4 and Tables 1, 2). One expected consequence of these *B. subtilis* DG101 attributes is the protection and increment of the beneficial portion of the indigenous gut flora (i.e., LAB). In this regard, we and others have demonstrated that *B. subtilis* is able to improve the growth and survival of LAB probiotics because of the proficiency of *B. subtilis* to form biofilms protecting LAB from the stressful gut environment (Zhang et al., 2013; VidyaLaxme et al., 2014; Yang et al., 2015; Yahav et al., 2018).

*B. subtilis* DG101 possesses an extra beneficial effect on the LAB population. It has been previously reported that the ability of *B. subtilis* natto to degrade starch (i.e., proficiency in amylase activity, Table 1C) provides LAB (unable to degrade starch) with the simple sugars needed for their metabolism and energy production (Horie et al., 2018). *B. subtilis* DG101 would be able to collaborate in food digestion and host metabolism because of the plethora of other hydrolytic exoenzymes with activity against macromolecules (e.g., proteins, lipids, and polysaccharides) present in the regular human diet (Table 1C). The beneficial gut-associated attributes of *B. subtilis* DG101 could be the reasons for the recognized efficacy of *B. subtilis* in alleviating GI discomfort, gas production, and bloating, and the improvement of the production of healthy polyunsaturated fatty acids in the gut (VidyaLaxme et al., 2014; Penet et al., 2021; Garvey et al., 2022).

*B. subtilis* DG101 shows the potential for the detoxification (i.e., bioremediation) of metal-contaminated foods or drinking water (Alotaibi et al., 2012) because of its proficiency in growing in the presence of toxic heavy metals (Cd_II_, Cr_VI_, and As_III_) and turning them into less toxic (transformation of Cr_VI_ and As_III_ into Cr_III_ and As_V_, respectively) or less available forms (absorbed Cd_II_) to impair the host intestinal absorption (Figures 3A,B).

*B. subtilis* DG101 possesses a high tolerance for growth at high temperatures in the presence of high salt concentrations (Table 1B), however, it lacks hemolytic activity (Table 1C and data not shown) and antimicrobial resistance (Table 1E). *B. subtilis* DG101 expresses a potent antimicrobial activity against pathogenic bacteria and fungi of medical concern (Table 2). The *in vitro* antimicrobial activity of *B. subtilis* DG101 (Table 2) correlates with the reported human protection against pathogenic *Salmonella enterica* and *Staphylococcus aureus* because of the production of natural lipopeptide antibiotics by *B. subtilis* (Piewngam et al., 2018; Piewngam and Otto, 2020).

Besides the demonstrated desirable attributes of *B. subtilis* DG101 (Figures 1–4 and Tables 2 and 1), this strain is able to survive the hostile environmental conditions (e.g., exposure to lysozyme, low pH, and bile salts) that it would encounter during transit through and colonization of the GI tract (Figure 5). In addition, *B. subtilis* DG101 was able to improve lipid homeostasis (e.g., decrease LDL and increase HDL levels) and the protein concentrations (e.g., albumin and globulins) present in the serum of animals fed with a supplement of the probiotic bacterium. These healthy lipid and serum protein patterns were maintained in animals exposed to nutritional stress (Figure 6). The *in vitro* and *in vivo* proficiencies of *B. subtilis* DG101 correlates well with the recently reported anti-type 2 diabetes mellitus and anti-obesity effects of *B. subtilis* DG01 in humans (Patterson et al., 2016; Cardinali et al., 2020; Rodriguez Ayala et al., 2022a, 2022b).

A high and regular human intake of nattō, but not total soy or isoflavonoids intake, has been associated with a lower risk of high blood pressure and stroke (Kim et al., 2008; Yan et al., 2017), and, importantly, with a significant reduction (~10%) of any cause of natural death, in particular cardiovascular disease (CVD) mortality (Zhang et al., 2003; Kokubo et al., 2007; Nagata et al., 2017; Yan et al., 2017; Katagiri et al., 2020; Rodriguez Ayala et al., 2021; Abe et al., 2022). The human beneficial compounds produced by *B. subtilis* DG101 and present in nattō food (e.g., nattoquinase, PQQ, vitamin K2, and lipopeptide antibiotics) and the results shown here, led us to hypothesize that the nattō-making bacterium *B. subtilis* DG101 can play an important role in the reported human healthy prolongevity effect of this traditional food (Rodriguez Ayala et al., 2017b, 2021). The proof of concept of this hypothesis was the antiage and healthy cognitive effects produced in *C. elegans* colonized by *B. subtilis* DG101 compared with the life expectancy of worms colonized by the non-probiotic bacteria *E. coli* OP50 or *B. subtilis* 168 (Figure 7).

## Data availability statement

The datasets presented in this study can be found in online repositories. The names of the repository/repositories and accession number(s) can be found below: https://www.ncbi.nlm.nih.gov/genbank/, OQ813498.

## Ethics statement

The animal study was approved by Comité de ética Universidad Nacional de Rosario. The study was conducted in accordance with the local legislation and institutional requirements.

## Author contributions

CL: Investigation, Methodology, Writing – original draft. FR: Investigation, Methodology, Writing – original draft. AG: Investigation, Methodology, Writing – original draft. LR: Investigation, Methodology, Writing – original draft. AN: Conceptualization, Methodology, Writing – original draft. RG: Conceptualization, Funding acquisition, Supervision, Validation, Writing – review & editing.

## Funding

This work was supported by FONCyT (Fondo para la Investigación Científica y Tecnológica) of Argentina and the Pew Latin-American Program in Biological Sciences (Philadelphia, United States).

## Conflict of interest

The authors declare that the research was conducted in the absence of any commercial or financial relationships that could be construed as a potential conflict of interest.

## Publisher’s note

All claims expressed in this article are solely those of the authors and do not necessarily represent those of their affiliated organizations, or those of the publisher, the editors and the reviewers. Any product that may be evaluated in this article, or claim that may be made by its manufacturer, is not guaranteed or endorsed by the publisher.

## Footnotes

^1^https://pasteur.uy/en/units/molecular-biology

^2^www.ncbi.nlm.nih.gov/blast

^3^http://rdp.cme.msu.edu/

^4^https://www.ncbi.nlm.nih.gov/nuccore/OQ813498

^5^https://asm.org/Articles/2022/February/Updating-Breakpoints-in-Antimicrobial-Susceptibili
